# *Anopheles culicifacies *breeding in brackish waters in Sri Lanka and implications for malaria control

**DOI:** 10.1186/1475-2875-9-106

**Published:** 2010-04-21

**Authors:** Pavilupillai J Jude, Sangaralingam Dharshini, Muthuladchumy Vinobaba, Sinnathamby N Surendran, Ranjan Ramasamy

**Affiliations:** 1 Department of Zoology, Faculty of Science, University of Jaffna, Jaffna, Sri Lanka; 2 Department of Zoology, Faculty of Science, Eastern University, Chenkaladi, Sri Lanka; 3 Institute of Medicine, Universiti Brunei Darussalam, Gadong, Brunei Darussalam

## Abstract

**Background:**

*Anopheles culicifacies *is the major vector of both falciparum and vivax malaria in Sri Lanka, while *Anopheles subpictus *and certain other species function as secondary vectors. In Sri Lanka, *An. culicifacies *is present as a species complex consisting of species B and E, while *An. subpictus *exists as a complex of species A-D. The freshwater breeding habit of *An. culicifacies *is well established. In order to further characterize the breeding sites of the major malaria vectors in Sri Lanka, a limited larval survey was carried out at a site in the Eastern province that was affected by the 2004 Asian tsunami.

**Methods:**

Anopheline larvae were collected fortnightly for six months from a brackish water body near Batticaloa town using dippers. Collected larvae were reared in the laboratory and the emerged adults were identified using standard keys. Sibling species status was established based on Y-chromosome morphology for *An. culicifacies *larvae and morphometric characteristics for *An. subpictus *larvae and adults. Salinity, dissolved oxygen and pH were determined at the larval collection site.

**Results:**

During a six month study covering dry and wet seasons, a total of 935 anopheline larvae were collected from this site that had salinity levels up to 4 parts per thousand at different times. Among the emerged adult mosquitoes, 661 were identified as *An. culicifacies s.l*. and 58 as *An. subpictus s.l*. Metaphase karyotyping of male larvae showed the presence of species E of the Culicifacies complex, and adult morphometric analysis the presence of species B of the Subpictus complex. Both species were able to breed in water with salinity levels up to 4 ppt.

**Conclusions:**

The study demonstrates the ability of *An. culicifacies *species E, the major vector of falciparum and vivax malaria in Sri Lanka, to oviposit and breed in brackish water. The sibling species B in the *An. subpictus *complex, a well-known salt water breeder and a secondary malaria vector in the country, was also detected at the same site. Since global warming and the rise in sea levels will further increase of inland brackish water bodies, the findings have significant implications for the control of malaria in Sri Lanka and elsewhere.

## Background

**Figure 1:**
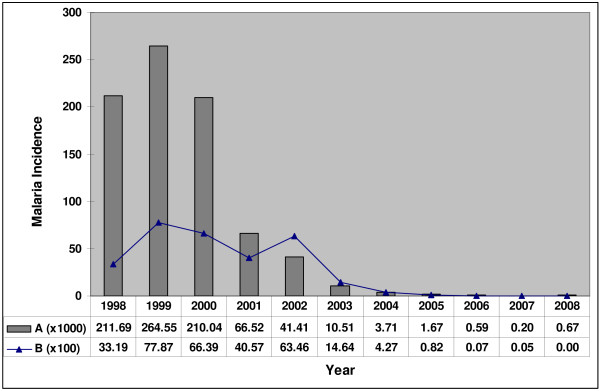
**Malaria incidence in the period 1998-2008 due to all species of malaria parasites (A) in Sri Lanka and (B) in Batticaloa district (adapted from data of Deputy Provincial Director for Health, Batticaloa)**.

*Anopheles culicifacies sensu lato (s.l.) *is well established as the major vector of both falciparum and vivax malaria in Sri Lanka, while *Anopheles subpictus s.l*. and certain other species function as secondary vectors [1-4]. Both vector species exist as a species complex in Sri Lanka and elsewhere in Asia [reviewed in [1]]. Only species B and E in the *An. culicifacies *complex of A-E have been detected in Sri Lanka with species E being incriminated as the major vector of falciparum and vivax malaria [5,6]. *An. culicifacies *species B and E of Sri Lanka can only be differentiated through Y-chromosome morphology [7]. All four known sibling species (A-D) of the *An. subpictus *complex are present in Sri Lanka [1]. However, only species B of *An. subpictus *has so far been shown to be a malaria vector in India [8]. The spraying of residual insecticides in households, larviciding breeding sites, the use of insecticide-treated and untreated bed nets, and early case detection and drug treatment are the major measures undertaken by the Ministry of Health to control malaria in the country. These measures have markedly reduced the incidence of malaria in Sri Lanka over the past decade (Figure 1) [9]. The Batticaloa district in the Eastern province of Sri Lanka, is located in the country's dry zone. Although it has traditionally been an area endemic for malaria, the Batticaloa district shows a decreasing malaria incidence in keeping with a country-wide trend (Figure 1). The Batticaloa district was badly affected by the Asian tsunami of December 2004. Batticaloa and other tsunami-affected eastern districts (Trincomalee and Ampara) of Sri Lanka had a high incidence of malaria before the tsunami [10], but the decrease in malaria incidence beginning 2004 showed that the tsunami did not increase malaria transmission in Batticaloa (Table 1) or elsewhere in the country (Figure 1).

**Table 1:** Malaria incidence in the district of Batticaloa in the Eastern province of Sri Lanka for the period 2001-2008 (source: Office of the Deputy Provincial Director of Health, Batticaloa).

Year	Number of blood film examined	Number of confirmed cases		^§^SPR	^†^API
				
		*P. vivax*	*P. falciparum*		
2001	52,149	3,771	286	7.8	7.98
2002	104,279	5,864	482	6.1	10.5
2003	109,787	1,348	116	1.3	2.5
2004	103,484	327	94	0.4	0.7
2005	92,817	73	09	0.08	0.14
2006	112,323	07	00	<0.01	0.06
2007	70,414	05	00	<0.01	0.08
2008	59,189	00	00	00	00

^§^SPR - slide positivity rate

^†^API - annual parasite index

The city of Batticaloa is surrounded by a lagoon contiguous with the Indian Ocean. The city is connected to the mainland via the Kallady Bridge. This article reports the brackish water breeding ability of species E of the Culicifacies complex and species B of the Subpictus complex, in the vicinity of Kallady Bridge and discusses the implications of the findings for the control of malaria.

## Methods

**Figure 2:**
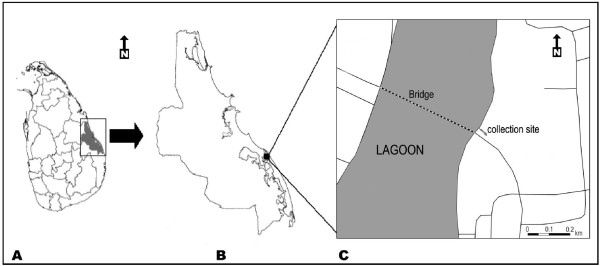
**Map showing the larval collection site in the Batticaloa district of Sri Lanka**. **A **- Sri Lanka; **B **- Batticaloa district; **C**- Batticaloa lagoon and sampled location.

Anopheline larvae were collected at a site 25 m away from the Kallady Bridge (Figure 2) within the city of Batticaloa (7°43'07.16" N; 81°42'31.28" E). The stagnant and permanent brackish water body where the collection was performed is connected to the large Batticaloa lagoon by a shallow channel. Mangroves and grass line the water body and the lagoon. Larval collections were carried out along the margin of the water body using an 8 cm diameter and 240 ml capacity dipper. In a single collection five dipper samples were taken from a one m^2 ^area and a total of ten collections were done covering the entire pool in every visit. Samples were collected fortnightly in the period from September 2009 to February 2010. The collected larvae along with water samples were transferred to the Eastern University Zoology laboratory in Batticaloa. The larvae were maintained under laboratory conditions (28 ± 2°C) to emerge as adults. Salinity (Salino meter, Atago, Japan), pH (Hanna Instruments, HI 98128, Romania) and dissolved oxygen (Hanna Instruments, HI 8043, Rumania) were measured in the water samples. The larvae and emerged adults were identified at the species level using standard published keys [11-13]. Morphometric characteristics were used to identify sibling species of *An. subpictus *[14]. Late third and early fourth male larvae from collected *An. culicifacies *were used to determine Y-chromosome morphology, and thereby identify *An. culicifacies *sibling species as described previously [5]

## Results

A total of 935 anopheline larvae were collected during the six month period. Among the emerged adults 661 were identified as *An. culicifacies *and 58 as *An. subpictus*. All the *An. subpictus *females were identified as sibling species B. Among fifteen randomly selected male *An. culicifacies *larvae, all five that could be successfully karyotyped possessed a submetacentirc Y-chromosome characteristic of sibling species E. The average measured values of salinity, pH and dissolved oxygen at larval collection site over the study period are shown in Table 2.

**Table 2:** Monthly anopheline collections and measured chemical parameters at Kallady.

Month	Chemical parameters			Number of adults identified	
		
	pH	Dissolved oxygen (mg/L)	Salinity (ppt)	*An. culicifacies*	*An. subpictus*
Sept, 2009	8.0	1.1	4	24	-
Oct, 2009	7.9	1.2	4	39	44
Nov, 2009	8.1	1.2	3	56	09
Dec, 2009	7.9	1.4	1	26	-
Jan, 2010	8.2	1.4	0	324	-
Feb, 2010	8.3	1.5	2	192	-

*Anopheles culicifacies *larvae were also collected from freshwater bodies within one km proximity to the Kalladay locality and brackish water bodies (salinity ranging from 2-3 parts per thousand) in at a different site called Raalkuli (nearly 110 km away from the present study location) in the tsunami-affected Trincomalee district of the Eastern province of Sri Lanka in the period from December 2009 to February 2010 (data not shown).

## Discussion

*Anopheles culicifacies s.l*., the major vector of malaria in the Indian subcontinent, is generally regarded to be intolerant of salinity [15,16], preferring to breed in newly-dug freshwater pits [15], domestic wells and pits used for plantation of coconuts and casurina [16] in India. However *An. culicifacies *larvae have reportedly been collected from concrete reservoir tanks containing brackish water containing 16-39% sea water in Oman, even though they survived best in freshwater [17]. Previous studies in Sri Lanka have reported that *An. culicifacies s.l*. breeds only in freshwater bodies (salinity <0.5 ppt) with high dissolved oxygen and is, therefore, confined to the riverine system of the country [18-20]. Vector control strategies through water management in the country are also based on the assumption that *An. culicifacies *only breeds in freshwater [21]. The brackish water breeding ability of sibling species B of Subpictus complex is, however, well established in India [22] and Sri Lanka [23].

Recent investigation on larval breeding sites of the members of Culicifacies complex in the country suggests that species E can exploit a wide range of fresh water breeding habitats [1,24]. However, the findings reported here are the first, from Sri Lanka, that demonstrate the ability of *An. culicifacies *species E, like species B of the *An. subpictus *complex, to breed in brackish waters. *Anopheles culicifacies *species is able to tolerate salinity changes in the same breeding ground over the period of time caused by monsoonal rain that began in late November 2009 and ended in January 2010. Possessing the ability to breed in brackish and fresh waters is a characteristic of *An. culicifacies *species E that is significant in the context of its traditional major role in malaria transmission in Sri Lanka.

Some members of the different anopheline species complexes show high salinity tolerance and this is associated with malaria transmission in coastal areas in different parts of the world. *Anopheles melas *and *Anopheles merus *within the *An. gambiae *complex are reported to be salt water species [25]. In the Farauti complex, *Anopheles farauti *No.1 is reported to be saline tolerant [26]. The *Anopheles sundaicus *complex in Asia, is composed of sibling species that breed in both fresh and saline waters [27].

It was postulated that salt water breeding species of *An. sundaicus s.l*. in south-east Asia may increase in numbers and contribute to greater malaria transmission in the wake of the Asian tsunami that produced significant intrusion of sea water into inland areas [28,29]. A larval survey carried out after the tsunami in south India revealed the invasion of *An. culicifacies *into the affected areas breeding in ground pools and coconut garden pits with salinity ranging from 2.3 to 7.1 ppt [30]. However, it was previously suggested that the tsunami-induced malaria risk was minimal in affected areas of Sri Lanka, since the major malaria vector *An. culicifacies s.l*. breeds only in freshwater bodies and *An. sundaicus s.l*. was absent in the country [31]. Although *An. culicifacies *species E and *An. subpictus *species B larvae are now reported from brackish water bodies in the tsunami affected areas, it is clear that this has not increased malaria transmission in the Eastern province to date. This is may be due to the continued implementation of effective malaria control measures in the country.

Whether *An. culicifacies *began breeding in brackish waters in the Batticaloa district as a result of the 2004 tsunami or only opportunistically utilizes brackish water as breeding sites, cannot be established because there have been no previous studies of larvae in such water bodies. *An. culicifacies *are also able to breed in fresh water bodies located less than one km away from Kallady. There are also no reliable data on the anopheline mosquito prevalence in the Batticaloa and other districts of the Eastern province and carrying out such a study was not previously feasible due to two decades of civil war in this part of the country. A limited study that investigated the human biting mosquitoes in Batticaloa during 1993-1994 detected only *An. subpictus s.l*. and not *An. culicifacies s.l*. [32].

Global warming consequent to increasing anthropogenic emissions of CO_2 _will lead to the melting of polar ice and a rise in sea levels. As a consequence, an increasing number of surface water bodies will become more saline in the coastal areas of many countries. These will provide fertile breeding grounds for the more salt -tolerant Anopheline vectors of malaria parasites and this poses a risk of increasing malaria transmission in coastal areas. Such an effect is likely to be compounded by increasing temperatures generally inducing a spread of the malaria vectors into greater latitudes in the temperate zones [33,34]. The ability of *An. culicifacies *species E to breed in brackish waters poses a particular problem for Sri Lanka as there are already many areas of the country with brackish surface waters e.g. the Jaffna and Puttalam districts in the north and the west of the island, respectively. Increased breeding of anophelines in selected local water bodies may provide the nuclei for initiating epidemics of malaria in Sri Lanka and elsewhere. For example, the rapid breeding of the relatively inefficient malaria vector *Anopheles annularis *in newly built irrigations canals in the north-central province of Sri Lanka was suggested to have triggered malaria epidemics towards the end of the last century [35]. The present findings, therefore, establish the need for systematic larval survey along the coastal belt of Sri Lanka to examine the prevalence of malaria vectors breeding in brackish waters.

## Conclusions

The present study reveals, for the first time in Sri Lanka, the ability of the potential malaria vector species E of the Culicifacies complex to breed in brackish waters. This developed ability may pose a threat of malaria transmission in coastal areas of Sri Lanka. Therefore, a systematic larval survey along the coastal belt of the country is warranted to devise an appropriate vector control measure by the Ministry of Health.

## Competing interests

The authors declare that they have no competing interests.

## Authors' contributions

SNS and RR conceived the study and wrote the manuscript. PJJ and SD performed the field collections MV provided logistic support. PJJ, SD, MV and SNS performed the identification and laboratory studies. All authors read and approved the final manuscript.
